# Transcriptome analysis reveals immune-related gene expression changes with age in giant panda (*Ailuropoda melanoleuca*) blood

**DOI:** 10.18632/aging.101747

**Published:** 2019-01-14

**Authors:** Lianming Du, Qin Liu, Fujun Shen, Zhenxin Fan, Rong Hou, Bisong Yue, Xiuyue Zhang

**Affiliations:** ^1^ Key Laboratory of Bio-resources and Eco-environment, Ministry of Education, College of Life Science, Sichuan University, Chengdu 610064, China; ^2^ Institute for Advanced Study, Chengdu University, Chengdu 610106, China; ^3^ College of Life Sciences and Food Engineering, Yibin University, Yibin 644000, China; ^4^ The Sichuan Key Laboratory for Conservation Biology of Endangered Wildlife, Chengdu Research Base of Giant Panda Breeding, Chengdu 610081, China; ^*^Equal contribution

**Keywords:** transcriptome, giant panda, immune system, ageing, gene expression

## Abstract

The giant panda (*Ailuropoda melanoleuca*), an endangered species endemic to western China, has long been threatened with extinction that is exacerbated by highly contagious and fatal diseases. Aging is the most well-defined risk factor for diseases and is associated with a decline in immune function leading to increased susceptibility to infection and reduced response to vaccination. Therefore, this study aimed to determine which genes and pathways show differential expression with age in blood tissues. We obtained 210 differentially expressed genes by RNA-seq, including 146 up-regulated and 64 down-regulated genes in old pandas (18-21yrs) compared to young pandas (2-6yrs). We identified ISG15, STAT1, IRF7 and DDX58 as the hub genes in the protein-protein interaction network. All of these genes were up-regulated with age and played important roles in response to pathogen invasion. Functional enrichment analysis indicated that up-regulated genes were mainly involved in innate immune response, while the down-regulated genes were mainly related to B cell activation. These may suggest that the innate immunity is relatively well preserved to compensate for the decline in the adaptive immune function. In conclusion, our findings will provide a foundation for future studies on the molecular mechanisms underlying immune changes associated with ageing.

## INTRODUCTION

The giant panda (*Ailuropoda melanoleuca*), an endangered species endemic to western China, has long been threatened with extinction due to human population expansion and habitat destruction [1, 2]. Recent research has shown that giant pandas are well adapted to a specialized bamboo diet via enigmatic gut microbiota and low energy expenditure [3–5]. Contrary to previous studies, the latest genome sequencing and resequencing and genome-wide genetic studies have demonstrated that the giant panda has a relatively high genetic diversity [6–8]. Over the years, extensive research has been conducted on genes of the major histocompatibility complex (MHC) in giant pandas and shown that DRB and DQA genes have low diversity but remain positive selection [9–11]. However, most of these studies focused on the adaptations and genetic diversity of giant pandas and little is known about the gene expression of immune-related genes.

Giant pandas are vulnerable to a variety of carnivore-specific and mammalian infectious and parasitic diseases that seriously threaten the giant panda populations [12–16]. For example, *Baylisascaris schroederi* is the most common parasite in wild and captive giant pandas and was responsible for half of panda deaths between 2001 and 2005 [15]. Likewise, canine distemper virus (CDV) is highly contagious and fatal and affects a wide range of mammals, including the giant panda [17]. In 2014–2015, an outbreak of CDV infection in giant pandas caused the death of five infected animals in two months [18].

The immune system is vulnerable to age-associated alterations, which accumulate to produce a progressive deterioration and lead to an increased incidence of infectious diseases [19]. Although aging is an inevitable biological process and a powerful risk factor for many diseases, the underlying molecular mechanisms that lead to generalized disease susceptibility are largely unknown [20, 21]. Aging has proven difficult to dissect in part due to its interactions with environmental influences, other genetic factors, and a large number of age-related diseases [22]. Immunosenescence, defined as immune changes with ageing, is an unavoidable life process and has been characterized in several species, such as humans [21, 23], mice [24], zebra finches [25] and wolves [26]. A comprehensive meta-analysis of age-related gene expression profiles indicated that signatures of aging most notably involve an overexpression of inflammation and immune response genes and an underexpression of genes associated with energy metabolism [27]. However, the effects of aging on giant panda have not been characterized, especially the age-related changes of immune system. Moreover, understanding the principles of giant panda immune system is vital for the development vaccines that can elicit protective immunity [28].

The aims of this study were to determine which genes and pathways show differential expression with age in giant panda blood tissues and to understand age-related alterations of the immune system. We used RNA-seq technology to identify age-related differentially expressed genes (DEGs) in giant panda blood samples and performed functional enrichment analysis for these DEGs. The collective data generated in this study may represent a valuable resource to enable further advancements in immunological research in giant pandas.

## RESULTS

### Transcriptome sequencing and assembly

RNA prepared from blood tissues of four giant pandas were subjected to RNA sequencing using Illumina HiSeq™ 2000. Together with the three giant panda transcriptomes from our previous study, we totally acquired approximately 187 million raw paired-end reads and 162 million remained after removing adaptor sequences and discarding low quality reads. The total length of the reads was about 35.8 gigabases (Gb). We aligned each of the seven short-read libraries onto the giant panda Ensembl reference genome (ailMel1) separately and found that an average of 19.86 million high-quality reads (85.68%) could be successfully mapped to the genome per sample. This included a mean of 19.31 million uniquely mapped reads (97.23%) per sample, indicating that the majority of the paired reads aligned correctly (Table 1). These high-quality reads were assembled into 56,543 genes, giving rise to 88,071 transcripts containing 46,091 (52.33%) transcripts that had more than one exon (Supplementary Figure 1). The comparison of similarities between the assembled transcripts and the Ensembl transcripts showed that only 2943 (3.34%) matched exactly with annotated intron chain and a total of 33,600 (38.15%) were identified as potentially novel isoforms of a predicted Ensembl transcript with at least one splice junction shared. The majority of transcripts (34,660, 39.35%) were annotated as intergenic transcripts and a small proportion of transcripts (7824, 8.88%) entirely fell into reference intron (Supplementary Table 1).

**Table 1:** Summary of sequencing and assembly of transcriptome

Sample	F02	F06	F18	F19	M05	M12	M21
Age	2	6	18	19	5	12	21
Group	Young	Young	Old	Old	Young	-	Old
Gender	Female	Female	Female	Female	Male	Male	Male
Read length (bp)	100	90	100	90	100	90	100
Raw read pairs	24,547,347	26,523,566	19,473,310	27,373,071	26,680,027	25,931,660	36,467,323
Total bases (Gb)	4.91	4.77	3.89	4.93	5.34	4.67	7.29
Clean read pairs	22,419,441	22,352,904	17,649,156	22,931,640	24,654,277	22,004,856	30,218,700
% of clean read pairs	91.33%	84.28%	90.63%	83.77%	92.41%	84.86%	82.87%
Mapped read pairs	19,423,023	20,089,734	15,064,083	20,316,914	21,831,830	19,131,977	27,080,383
% of mapped reads	86.63%	89.88%	85.35%	88.60%	88.55%	86.94%	89.61%
Uniquely mapped read pairs	18,975,652	19,663,458	14,711,801	19,892,536	21,380,756	18,621,171	26,492,780
% of uniquely mapped reads	97.70%	97.88%	97.66%	97.91%	97.93%	97.33%	97.83%

### Identification of age-related differentially expressed genes

**Figure 1:**
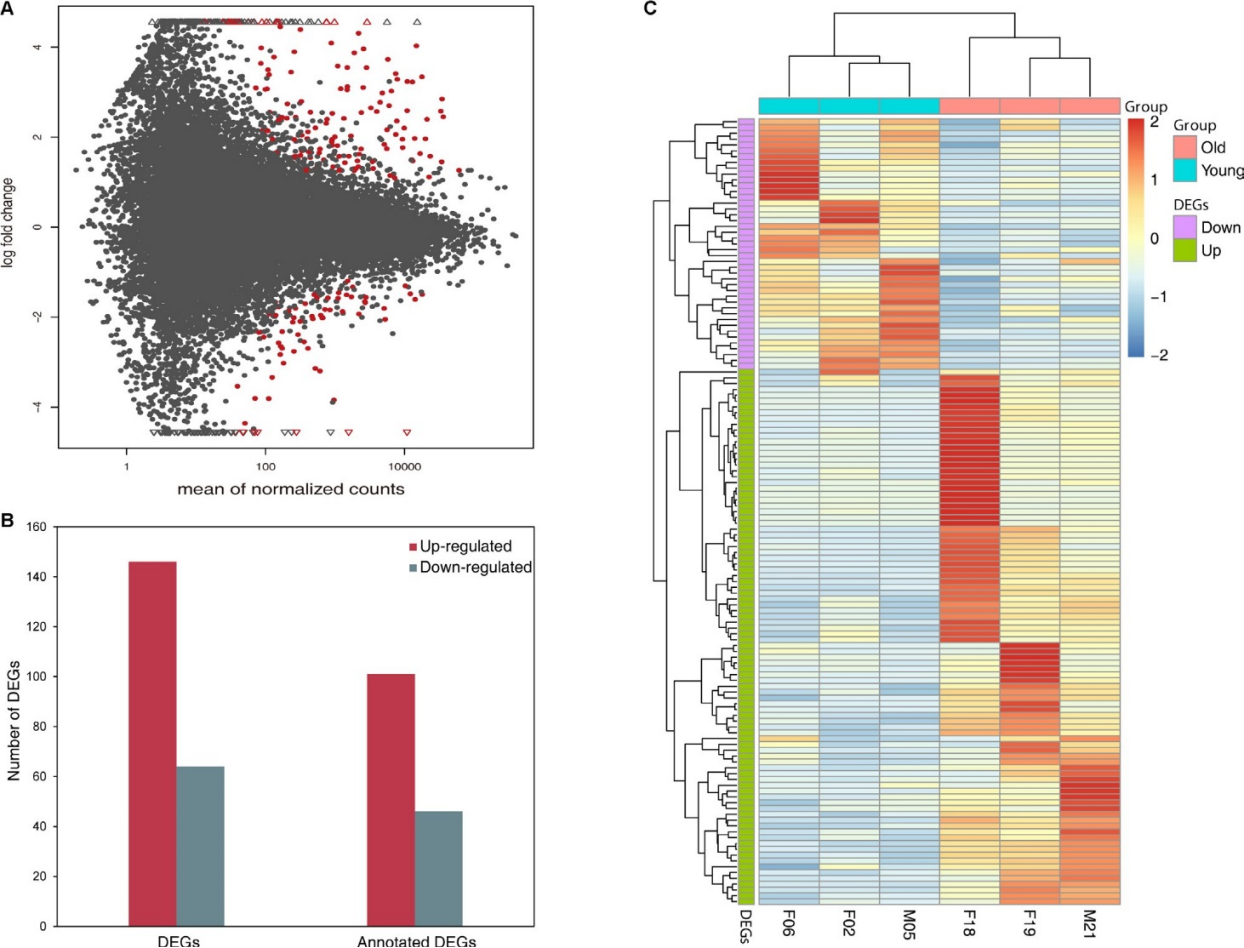
**Differentially expressed genes in old group compared to young group.** (**A**) MA plot showing the distribution of gene expression plotted against log2 fold change for each gene. Red dots indicate differentially expressed genes (FDR ≤ 0.05), black dots indicate non-differentially expressed genes. (**B**) Number of up- and down-regulated DEGs and annotated DEGs. (**C**) Heat map plot of DEGs using TPM expression value of genes by adopting hierarchical clustering method. The expression values of six individual are presented after being centered and scaled in the row direction. Each column represents a specimen and each row represents a gene. Red color indicates genes that were up-regulated and blue color indicates genes that were down-regulated.

The samples were categorized by giant panda age into a young group (i.e. 2, 5, 6 years) and an old group (i.e. 18, 19, 21 years) according to an earlier study [29]. The giant panda aged 12 years (i.e. a middle-aged adult) was not used to identify DEGs due to a lack of biological replicates. In total, 210 genes were found to show significant differential expression in giant panda blood samples between the young group and old group, after adjusting for confounding factors such as sex and library (Figure 1A, Supplementary Table 2). Among those DEGs, 146 genes were up-regulated while 64 genes were down-regulated in the old group compared to young group, and the annotated up- and down-regulated genes were 113 and 49, respectively (Figure 1B). In addition, we conducted a hierarchical clustering of DEGs across six samples. Genes that were up-regulated clustered into one group and the genes that were down-regulated clustered into another group. Similarly, young samples were clustered into one group and old samples were clustered into another group (Figure 1C), which also highlights the expression level of DEGs in the old group compared to the young group. To determine the relationship of these DEGs with age, we compared all the detected DEGs to age-related genes in purified human immune cells, human peripheral blood and wolf leukocytes. We found that 23 DEGs were presented in human studies [21] and only one DEG was presented in wolf study [26]. The 20 most significantly up- and down-regulated genes are shown in Tables 2 and 3. We also found that 2 MHC class I genes (LOC105855951, LOC102746195) and 3 MHC class II genes (LOC100464025, LOC101370503, LOC100464780) were both up-regulated in the old group.

**Table 2:** The top 20 most significantly up-regulated DEGs with annotation in old group compared with young group

Gene ID	Gene symbol	Gene name	Log2 fold change	FDR
MSTRG.14771	SERPING1	serpin family G member 1	9.367	4.03E-28
MSTRG.6700	BATF2	basic leucine zipper ATF-like transcription factor 2	5.000	7.87E-15
MSTRG.12698	RSAD2	radical S-adenosyl methionine domain containing 2	3.579	2.47E-12
MSTRG.27622	LOC105855951	HLA class I histocompatibility antigen, B-37 alpha chain-like	2.360	1.20E-11
MSTRG.14769	SMTNL1	smoothelin like 1	4.390	7.55E-11
MSTRG.11289	LOC100472013	hemoglobin subunit alpha	2.577	1.93E-08
MSTRG.16263	AXL	AXL receptor tyrosine kinase	2.552	1.93E-08
MSTRG.21559	NINJ1	ninjurin 1	2.981	3.29E-08
MSTRG.19528	MX2	MX dynamin like GTPase 2	2.395	6.92E-08
MSTRG.25578	LOC100464206	T-cell-specific guanine nucleotide triphosphate-binding protein 1-like	3.235	1.34E-07
MSTRG.9032	IDO1	indoleamine 2,3-dioxygenase 1	5.418	1.94E-07
MSTRG.17793	CXCL10	C-X-C motif chemokine ligand 10	6.606	2.53E-07
MSTRG.22824	DDX58	DExD/H-box helicase 58	3.053	3.27E-07
MSTRG.10367	C1qb	Complement C1q subcomponent subunit B	4.308	5.51E-07
MSTRG.1957	HERC5	E3 ISG15—protein ligase HERC5	2.568	5.61E-07
MSTRG.25405	ISG15	ISG15 ubiquitin-like modifier	4.028	1.36E-06
MSTRG.19832	HBB	Hemoglobin subunit beta	2.847	1.65E-06
MSTRG.25584	LOC100463703	ncRNA	3.226	1.99E-06
MSTRG.17944	BCL2L14	BCL2 like 14	8.036	1.99E-06
MSTRG.25574	LOC100463955	T-cell-specific guanine nucleotide triphosphate-binding protein 1	3.412	2.46E-06

**Table 3:** The top 20 most significantly down-regulated DEGs with annotation in old group compared with young group

Gene ID	Gene symbol	Gene name	Log2 fold change	FDR
MSTRG.23360	C4BPA	complement component 4 binding protein alpha	−2.760	3.70E-06
MSTRG.28599		Ig gamma-3 chain C region	−2.049	9.32E-06
MSTRG.25526	PANDA_020697	T-cell receptor gamma chain C region 5/10–13	−2.037	1.74E-05
MSTRG.19412	COX6A2	cytochrome c oxidase subunit 6A2	−2.692	5.82E-05
MSTRG.20201	LOC105863300	extensin-3-like	−1.957	7.14E-05
MSTRG.13555	AATK	apoptosis associated tyrosine kinase	−1.652	1.25E-04
MSTRG.17977	PTGS2	prostaglandin-endoperoxide synthase 2	−2.015	1.25E-04
MSTRG.24469	DUSP23	Dual specificity protein phosphatase 23	−3.336	1.61E-04
MSTRG.27223	TRG	uncharacterized TRG	−2.322	2.41E-04
MSTRG.10093	Pol	LINE-1 retrotransposable element ORF2 protein	−6.072	2.78E-04
MSTRG.23358	LOC100483222	apolipoprotein R	−3.138	3.44E-04
MSTRG.22445	LOC100468283	leukocyte immunoglobulin-like receptor subfamily A member 6	−3.810	4.58E-04
MSTRG.26753	LOC105234997	CMRF35-like molecule 7	−2.650	8.34E-04
MSTRG.19414	LOC100475588	integrin alpha-D	−2.016	1.01E-03
MSTRG.14860	LOC101551452	tripartite motif-containing protein 38-like	−16.681	1.01E-03
MSTRG.14272	IGKC	Immunoglobulin kappa constant	−1.494	1.09E-03
MSTRG.10307	PRDM16	PR/SET domain 16	−2.829	1.15E-03
MSTRG.22018	BCAR1	BCAR1, Cas family scaffolding protein	−3.197	1.28E-03
MSTRG.9362	SLC4A11	solute carrier family 4 member 11	−2.013	1.36E-03
MSTRG.5838	Gab2	GRB2 associated binding protein 2	−6.720	1.42E-03

### Gene ontology enrichment analysis of differentially expressed genes

**Figure 2:**
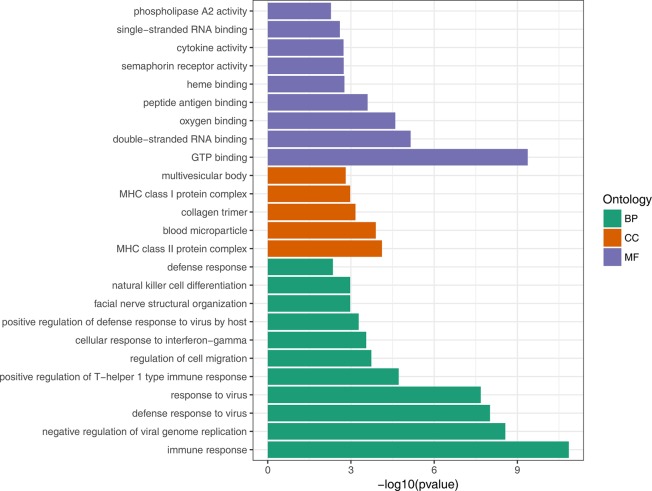
GO enrichment analysis of up-regulated DEGs

**Figure 3:**
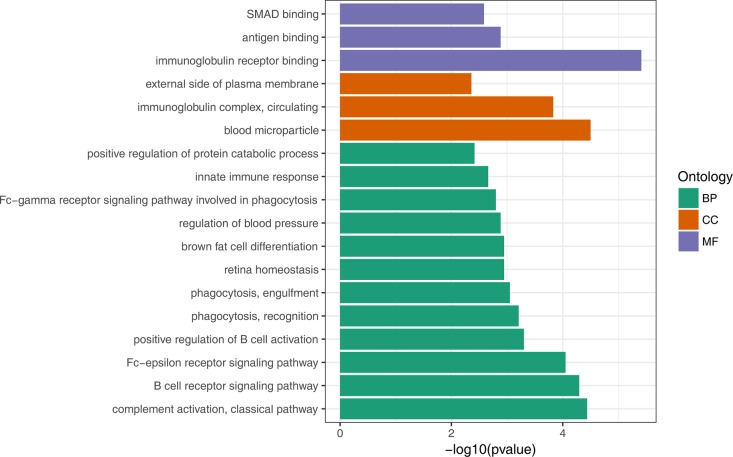
GO enrichment analysis of down-regulated DEGs

To gain insights into the biological roles of the DEGs, we performed a GO categories enrichment analysis. Only the annotated DEGs were selected and tested against the background set of all genes with GO annotation. We examined the GO categories separately. We found that for up-regulated genes with age, almost all the significantly enriched GO terms in biological process category were associated with the immune system, such as, immune response (GO:0006955), negative regulation of viral genome replication (GO:0045071), and defense response to virus (GO:0051607). MHC class II protein complex (GO:0042613) and GTP binding (GO:0005525) were the most significantly enriched GO term in the cellular component and molecular function categories, respectively (Figure 2, Supplementary Table 3). For down-regulated genes with age, the most significantly enriched biological process GO terms were complement activation, classical pathway (GO:0006958), B cell receptor signaling pathway (GO:0050853) and Fc-epsilon receptor signaling pathway (GO:0038095) (Figure 3, Supplementary Table 4). Interestingly, several up-regulated genes were enriched in retina homeostasis (GO:0001895) and regulation of blood pressure (GO:0008217), which might be related to age.

### Pathway enrichment analysis of differentially expressed genes

**Figure 4:**
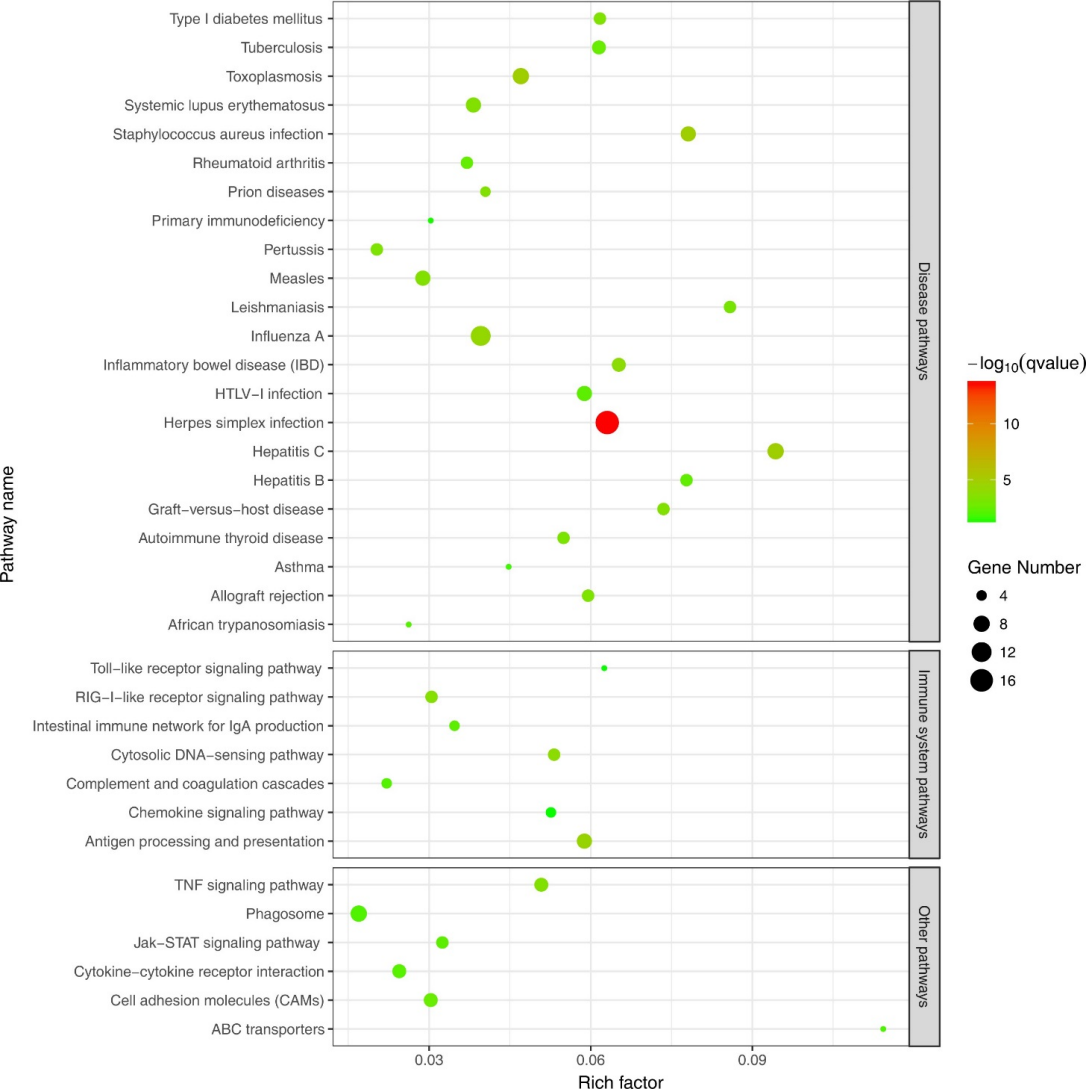
KEGG pathway enrichment analysis of up-regulated DEGs

To further evaluate the biological significance of the DEGs, we also performed KEGG pathway enrichment analysis. Hypergeometric tests with a *P* value cutoff of 0.05 was used as the criteria for pathway detection. We found that up-regulated genes were significantly enriched in 35 pathways, among which 22 pathways were disease related and 7 pathways were immune related. However, down-regulated genes were not significantly enriched to any pathways and this may be because many down-regulated genes were not annotated in KEGG pathways. Figure 4 illustrates the result of pathway enrichment of up-regulated genes, in which antigen processing and presentation (ko04612), cytosolic DNA-sensing pathway (ko04623) and RIG-I-like receptor signaling pathway (ko04622) were most significantly enriched.

### Protein-protein interaction network of differentially expressed genes

**Figure 5:**
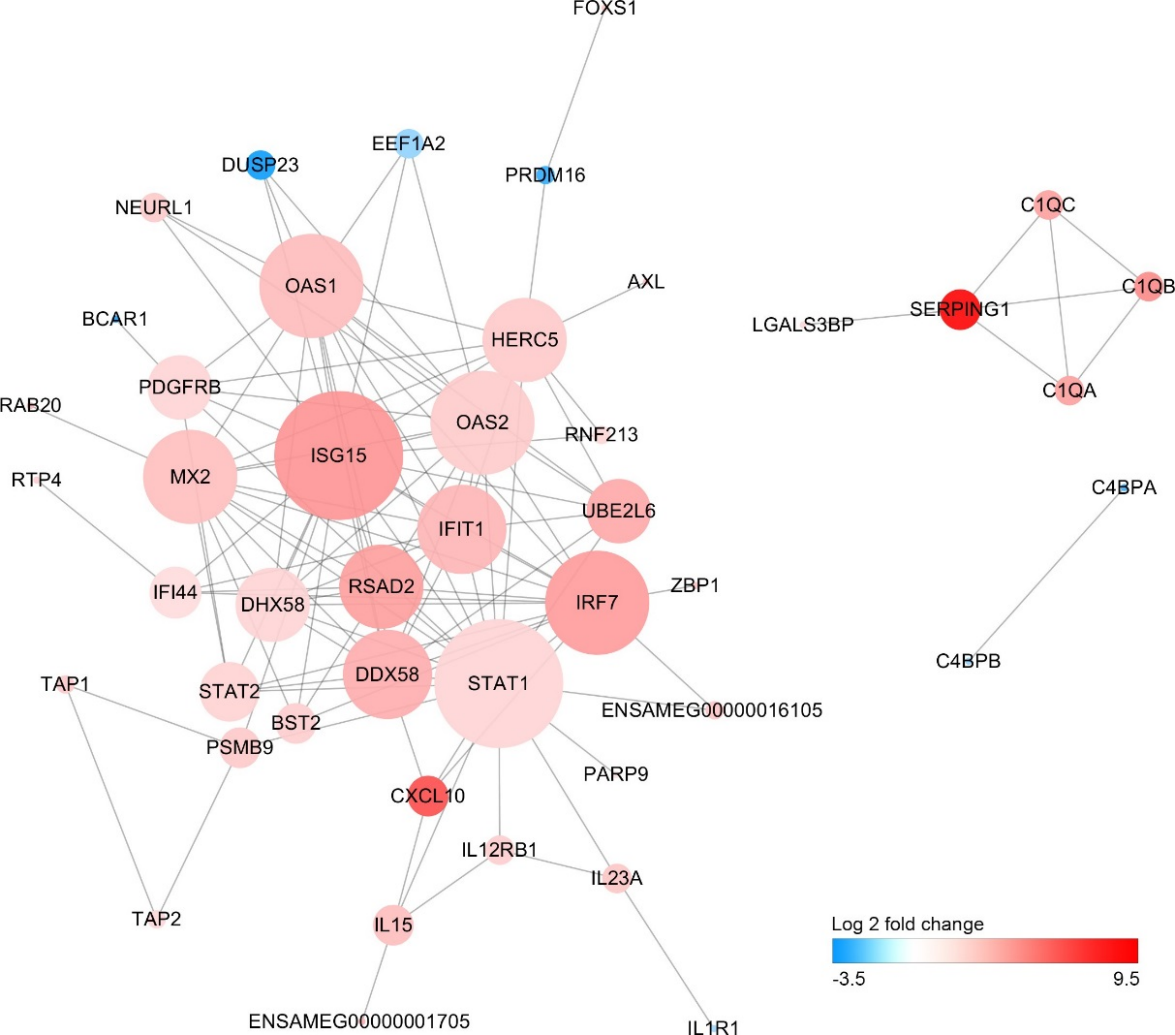
**Protein-protein interaction network of differentially expressed genes.** Size of the node is proportional to the number of DEGs interacted with it, and color of node represents Log2FoldChange in expression levels of DEGs between old and young giant pandas.

We have performed a protein-protein interaction analysis of all DEGs. A total of 122 interactions between 36 DEGs were extracted from the STRING database. Interferon-stimulated gene 15 (ISG15), signal transducer and activator of transcription 1 (STAT1), interferon regulatory factor 7 (IRF7) and DExD/H-Box Helicase 58 (DDX58) that play important roles in response to pathogen invasion were at key position of the interaction network (Figure 5).

## DISCUSSION

In a previous study, we have characterized giant panda blood transcriptome and identified 15 immune pathways where more than 70% of the total known genes were mapped by assembled transcripts [30]. In the present study, we still applied the widely used RNA-seq approach to highlight significant DEGs in blood between young and old giant pandas. The previous work has proved that age had a broad impact on gene expression levels, whereas sex had very minimal effects on gene expression patterns [26]. Therefore, it is appropriate to investigate age-related changes in giant panda by sequencing the blood transcriptome. To the best of our knowledge, this is the first study to assess the impact of age on immune-related gene expression.

The assembly results unfortunately underlined the incompleteness of the giant panda genome annotation of current version, and the need for further studies to decipher its complexity. We have provided a valuable resource for improvement of genome assembly and annotation. For example, a recent study has improved the genome assembly and identified novel transcripts by using transcriptomes from 12 giant panda tissues, excluding blood [31]. Another major outcome of transcriptome sequencing is the ability to identify DEGs between different conditions [32]. We identified 210 DEGs, which is much lower than the 625 and 1497 DEGs found in wolves [26] and humans [21], respectively. This may suggest that age-related alterations of gene expression are species-specific [33]. The most significantly up-regulated gene SERPING1, as a signature of mammalian aging, encodes the complement component 1 (C1) inhibitor [27]. The C1 inhibitor’s main function is the inhibition of several different proteases in the complement, contact, coagulation and fibrinolytic systems and has been reported to be related to an increased risk of several age-related human diseases [34–36]. The most significantly down-regulated gene C4BPA encodes the alpha-subunit of complement 4 binding protein. C4BPA is a key regulator of the complement system and has been considered a major factor in the age-related decline of the immune response due to decreased expression [37].

We identified age-related DEGs that may be critical in the progression of the giant panda aging process, such as ISG15, STAT1, IRF7. ISG15 is an innate immune and interferon-induced protein that plays a central role in response to viral infection [38]. ISG15 is also a stress-response gene that has been implicated as a tumor suppressor and contributor to inflammatory responses [39]. ISG15 expression was regulated by telomere length and increased with shortening telomeres caused by aging [40]. STAT1 carries tumor suppressor functions and regulates various cellular activities, such as apoptosis, angiogenesis, invasion and evasion of the immune system [41, 42]. Previous studies indicated that loss of STAT1 expression has been implicated in the pathobiology of various types of human cancer [41]. IRF7 has been discovered as the crucial regulator of type I interferons against pathogenic infections and plays important roles in immune modulation and regulation of oncogenesis and apoptosis [43]. Our results showed ISG15 can interact with both STAT1 and IRF7 and other antiviral related genes. It is possible that giant panda aging increased expression of ISG15 result in regulating innate immunity by interacting with genes involved in anti-viral responses. Furthermore, the invasive viruses can lead to activation of IRFs and induction of interferons (IFNs) resulting in antiviral IFNs signal through the JAK/STAT pathway to induce ISG production [44].

Aging is associated with functional integrity of the immune system declines leading to increased susceptibility to aging diseases [45]. Both GO and KEGG enrichment analysis showed that up-regulated genes were mainly involved in the immune response, particularly defense against viruses. The involved genes, such as IFIT5, BST2 and OAS1, were closely related to the innate immunity response [46–48]. In contrast, the down-regulated genes were implicated in the B cell receptor signaling pathway and positive regulation of B cell activation. In accordance with observations in wolf blood transcriptome with age, down-regulated genes were mainly associated with B cells and up-regulated genes were significantly associated with innate immunity [26]. Although, both innate and adaptive immune responses are affected by the aging process, the adaptive response seems to be more affected by age-related changes [49]. However, contrary to the previous results observed in the wolf [26], MHC class I and II genes were up-regulated with age in our study. In addition to their central role in adaptive immunity, both MHC class I and II genes can regulate Toll-Like Receptor to mediate the innate immune response [50, 51]. Taken together, these suggest the possibility that the aging immune system maintains a persistently activated innate immune response to compensate for the decline in the adaptive immune function [52].

Vaccines appear as one of the most efficient medical interventions against infectious diseases [53] and have been also used to assist giant pandas to defend against CDV infections [54, 55]. However, the vaccines for giant pandas do not elicit consistent antibody titers [56]. One explanation may be that the attenuated vaccine is inadequate to stimulate giant pandas to produce a high level of antibodies against CDV [55]. Another possible explanation might be aging-associated declines in immune system function. It is clear that the decline in adaptive immunity leads to dramatically reduced vaccine responses and vaccine longevity in older individuals [57]. We have noted that the immune response genes, such as IGHV3-23 and IGKC, that participate in the antigen recognition and B cell activation were down-regulated with ageing. This may lead to a decline of adaptive immunity resulting in decreased response to vaccines in giant pandas. In addition, the decreased ability of the adaptive immune system is closely related with the decline in production of naive lymphocytes and the accumulation of functionally impaired memory lymphocytes during aging [58].

In conclusion, we identified 210 genes which differentially expressed between old and young giant pandas. The up-regulated genes with age were mainly involved in the innate immune response, particularly in defense against virus. Instead, the down-regulated genes with age were mainly related to B cell activation. This suggests that innate immunity is relatively well preserved in aged giant pandas, although adaptive immunity deteriorates due to a decline of naive lymphocytes and the expansion of incompetent memory lymphocytes. Moreover, ISG15, STAT1, IRF7 and DDX58 were identified as the hub genes that play a pivotal role in response to viral infection and their expression were up-regulated with ageing. This work provides insight into gene expression changes associated with aging and will provide a foundation for future studies on the molecular mechanisms underlying immune changes associated with ageing.

## MATERIALS AND METHODS

### Sample collection

Blood samples were collected during a routine examination from four captive giant pandas at the Chengdu Research Base of Giant Panda Breeding in Chengdu, China. The other three giant panda blood transcriptomes were obtained from our previous study in NCBI SRA (project Accession no. SRP041998). Samples collection and utility protocols were approved by the Chengdu Institute of Biology Animal Use Ethics, which is responsible for the Chengdu Research Base of Giant Panda Breeding. The fresh blood samples were immediately stored in RNAlater (Ambion Inc., Austin, TX, USA). Total RNA was extracted using TRIzol reagent (Invitrogen, Carlsbad, CA, USA) following the manufacturer’s protocol and treated with RNase-free DNase I.

### Library preparation and sequencing

A total amount of 3 μg RNA per sample was used as input material for the RNA sample preparations. Sequencing libraries were generated using NEBNext^®^ Ultra™ RNA Library Prep Kit for Illumina^®^ (NEB, USA) following manufacturer’s recommendations and index codes were added to attribute sequences to each sample. Briefly, mRNA was purified from total RNA using poly-T oligo-attached magnetic beads. Fragmentation was carried out using divalent cations under elevated temperature in NEBNext First Strand Synthesis Reaction Buffer (5X). First strand cDNA was synthesized using random hexamer primer and M-MuLV Reverse Transcriptase (RNase H^−^). Second strand cDNA synthesis was subsequently performed using DNA Polymerase I and RNase H. Remaining overhangs were converted into blunt ends via exonuclease/polymerase activities. After adenylation of 3’ ends of DNA fragments, NEBNext Adaptor with hairpin loop structure were ligated to prepare for hybridization. In order to select cDNA fragments of 150~200 bp in length, the library fragments were purified with AMPure XP system (Beckman Coulter, Beverly, USA). Then 3 μl USER Enzyme (NEB, USA) was used with size-selected, adaptor-ligated cDNA at 37° C for 15 min followed by 5 min at 95° C before PCR. Then PCR was performed with Phusion High-Fidelity DNA polymerase, Universal PCR primers and Index (X) Primer. Lastly, PCR products were purified (AMPure XP system) and library quality was assessed on the Agilent Bioanalyzer 2100 system. The library preparations were sequenced on an Illumina Hiseq 2000 platform and 100 bp paired-end reads were generated.

### Quality control and transcriptome assembly

The giant panda genome assembly ailMel1 and reference annotation were downloaded from Ensembl v83 (www.ensembl.org). In order to obtain high quality reads, we employed NGS QC Toolkit [59] with stringent criteria (high-quality paired reads with more than 90% of bases with *Q*-value ≥ 20 were retained) to remove the low-quality paired-end reads or reads containing adaptors. High-quality paired-end reads from seven libraries were mapped on giant panda reference genome using HISAT [60], separately. Each of the alignment output files were assembled into separate transcriptomes using StringTie [61], which produces a transcript GTF file. The transcript GTF files produced after assembling were merged together to generate a complete transcript annotation file using StringTie with–merge option.

### Functional annotation

The merged assemblies were compared using the Cufflinks [62] inclusive utility Cuffcompare with the Ensembl annotations to find known and novel genes and isoforms, as well as transcripts expressed from intergenic regions. Only the genes with “=” and “j” class code were considered to be known genes. The GO annotations of these genes were extracted from Ensembl BioMart (http://www.ensembl.org/biomart/martview). The unknown gene sequences were searched against Swiss-Prot and NCBI non-redundant (NR) database using BLASTX [63] with a typical cutoff E-value of 1E-5. Gene ontology (GO) was applied with the Blast2GO [64] to obtain annotation of unknown genes.

### Gene expression estimation and differentially expressed genes analysis

We used the merged GTF file as the reference annotation file to guide the assembly process. StringTie was performed to estimate the genes expression level with -G, -b and -e options. The expression value of TPM (transcripts per million) and raw read counts for each gene were extracted from above result. Differential expression analysis was performed using DESeq2 [65] that takes the raw read count as the input. The results of all statistical tests were corrected for multiple testing with the Benjamini-Hochberg false discovery rate (FDR ≤ 0.05) and an absolute value of log 2 fold change ≥ 1 was used to determine significant differences in gene expression.

### Gene enrichment analysis

To functional classify the DEGs, we performed GO enrichment analysis using clusterProfiler [66] and searched for over-represented GO terms in three categories, namely biological process, molecular function and cellular component. We also performed KEGG pathway enrichment analysis using KOBAS [67]. An FDR cut-off of 0.05 was considered to be significantly enriched.

### Protein-protein interaction network analysis

Protein-protein interaction networks of DEGs were constructed on the basis of the STRING [68] protein interaction database. Cytoscape [69] was used to visualize the protein-protein interaction network for DEGs, and find the hub genes.

## SUPPLEMENTARY MATERIAL

Supplementary Figure

Supplementary Table 1

Supplementary Table 2

Supplementary Table 3

Supplementary Table 4
