# Supplementary material for: Transcriptome analysis reveals immune-related gene expression changes with age in giant panda (Ailuropoda melanoleuca) blood
Source: Aging (Albany NY). 2019 Jan 14;11(1):249–62. doi: 10.18632/aging.101747 (PMC6339791; doi:10.18632/aging.101747)
Supplement: Supplementary Figure [file aging-11-101747-s001.pdf]

SUPPLEMENTARY FIGURE

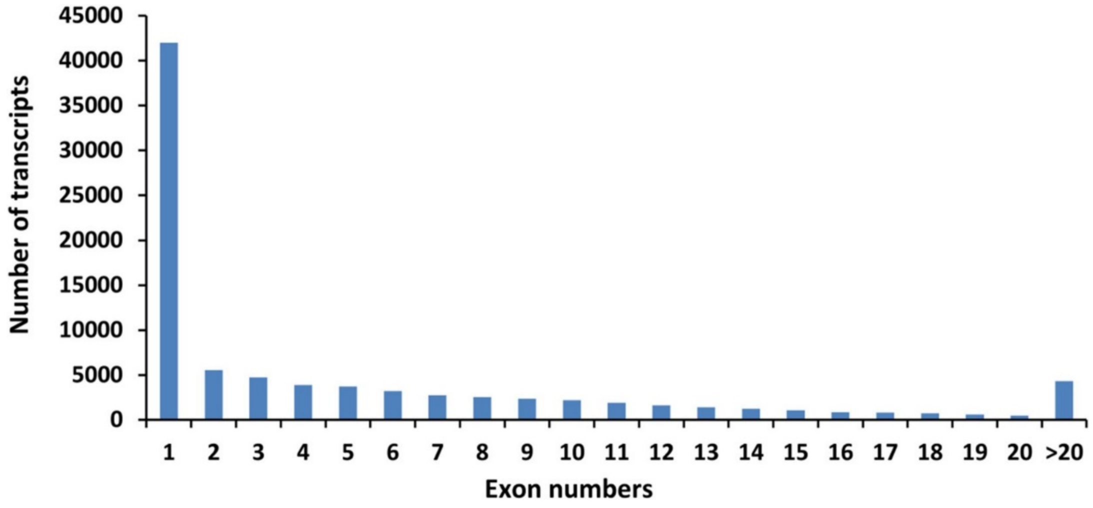

Supplementary Figure 1. Distribution of exon numbers of assembled transcripts.
