# Supplementary material for: Transcriptome analysis reveals immune-related gene expression changes with age in giant panda (Ailuropoda melanoleuca) blood
Source: Aging (Albany NY). 2019 Jan 14;11(1):249–62. doi: 10.18632/aging.101747 (PMC6339791; doi:10.18632/aging.101747)
Supplement: Supplementary Table 1 [file aging-11-101747-s002.pdf]

SUPPLEMENTARY TABLE

Supplementary Table 1. Classification of the transcripts identified in the blood samples in relation to the Ensembl annotated giant panda genes based on the Cuffcompare tool of Cufflinks.

Cuffcompare code	Number of transcripts	% of transcripts	Description
u	34660	39.35	Unknown, intergenic transcript
j	33600	38.15	Potentially novel isoform (fragment): at least one splice junction is shared with a reference transcript
i	7824	8.88	A transfrag falling entirely within a reference intron
=	2943	3.34	Complete match of intron chain
o	2611	2.96	Generic exonic overlap with a reference transcript
p	1967	2.23	Possible polymerase run-on fragment (within 2Kbases of a reference transcript)
x	1849	2.1	Exonic overlap with reference on the opposite strand
c	1486	1.69	Contained
e	1098	1.25	Single exon transfrag overlapping a reference exon and at least 10 bp of a reference intron, indicating a possible pre-mRNA fragment.
s	33	0.04	An intron of the transfrag overlaps a reference intron on the opposite strand (likely due to read mapping errors)
total	88071	100	
